# When holding your horses meets the deer in the headlights: time-frequency characteristics of global and selective stopping under conditions of proactive and reactive control

**DOI:** 10.3389/fnhum.2014.00994

**Published:** 2014-12-10

**Authors:** Christina F. Lavallee, Marie T. Meemken, Christoph S. Herrmann, Rene J. Huster

**Affiliations:** ^1^Experimental Psychology Laboratory, European Medical School, Department of Psychology, University of OldenburgOldenburg, Germany; ^2^Department of Neurology, Max Planck Institute for Human Cognitive and Brain SciencesLeipzig, Germany; ^3^Research Centre Neurosensory Science, University of OldenburgOldenburg, Germany

**Keywords:** stop-signal task, inhibition, selectivity, reactive inhibition, proactive inhibition, EEG, time-frequency analysis

## Abstract

The ability to inhibit unwanted thoughts or actions is crucial for successful functioning in daily life; however, this ability is often impaired in a number of psychiatric disorders. Despite the relevance of inhibition in everyday situations, current models of inhibition are rather simplistic and provide little generalizability especially in the face of clinical disorders. Thus, given the importance of inhibition for proper cognitive functioning, the need for a paradigm, which incorporates factors that will subsequently improve the current model for understanding inhibition, is of high demand. A popular paradigm used to assess motor inhibition, the stop-signal paradigm, can be modified to further advance the current conceptual model of inhibitory control and thus provide a basis for better understanding different facets of inhibition. Namely, in this study, we have developed a novel version of the stop-signal task to assess how preparation (that is, whether reactive or proactive) and selectivity of the stopping behavior effect well-known time-frequency characteristics associated with successful inhibition and concomitant behavioral measures. With this innovative paradigm, we demonstrate that the selective nature of the stopping task modulates theta and motoric beta activity and we further provide the first account of delta activity as an electrophysiological feature sensitive to both manipulations of selectivity and preparatory control.

## Introduction

The role of executive functions and cognitive control in successful human behavior and adaptation is crucial. One of these cognitive functions, inhibition, allows humans the ability to stop unwanted behaviors, as well as suppress mental representations. Its impairment is the cornerstone of many psychological disorders (Chamberlain and Sahakian, 2007), such as attention deficit hyperactivity disorder (ADHD; Aron and Poldrack, 2005; Senderecka et al., 2012) and schizophrenia (Zandbelt et al., 2011; Hughes et al., 2012), among others (Chamberlain et al., 2006; Bari and Robbins, 2013). The stop-signal task is a particularly popular paradigm utilized to assess reactive inhibitory mechanisms, in which participants must make a motor response (i.e., button press) to go-signals on go trials and must withhold this response when infrequently presented stop-signals, appearing after a delay (stop-signal delay “SSD”), are displayed on stop trials. This basic version of the stop-signal paradigm is thought to elicit outright or reactive stopping to an unexpected stimulus; however, it has recently been proposed that, based on the dual mechanisms of control (DMC) framework (Braver, 2012), cognitive control operates via two modes of preparation: proactively and reactively (Aron, 2011). When provided goal-relevant information, subjects actively maintain this information prior to the occurrence of cognitively demanding events under proactive control. Whereby proactive control is developed based on foreknowledge of upcoming demands and/or the subject’s current goals, reactive control, as employed in a typical stop-signal paradigm, is recruited immediately after the detection of a high-interference event (Braver, 2012). Proactive control entails having advanced information of the upcoming response that must be stopped (Aron and Verbruggen, 2008), which can be accomplished via cueing (Swann et al., 2013). That is, under proactive control conditions, subjects adapt their behavior in a goal-oriented fashion, similar to the holding your horses analogy of inhibition; whereas, the rather abrupt reactive control mode is more akin to a deer in the headlights metaphor, which refers to the immediate stopping in reaction to an unexpected stimulus.

In addition to preparatory mode of control, whether reactive or proactive, the mechanism of the stopping behavior may also differ. Mechanistically, under stopping conditions, inhibition can be employed either globally (i.e., all behaviors) or selectively (i.e., a subset of behaviors) and these mechanisms are also considered important for developing a more encompassing model of inhibition. Standard stopping is proposed to engage global suppression whereas behaviorally selective stopping (that is, stopping one response and executing another) is proposed to engage a selective suppression mechanism, as shown via transcranial magnetic stimulation (TMS) studies (Majid et al., 2012). Furthermore, the degree of selectivity was influenced by advanced information, suggesting that the nature of selective stopping is influenced by preparatory information. Thus, this demonstrates a greater need to develop a paradigm incorporating global and selective stopping conditions with and without the presence of additional information (i.e., foreknowledge) on which response should be stopped. The relevance for everyday life becomes clear when considering that most situations do not simply require global or reactive stopping (as measured in classic versions of the stop-signal task), such as immediately having to halt before crossing the street when you notice a speeding car running a red light. Many situations require some selectivity and may be shaped by the context of the environment or by personal goals. For example, when playing sports such as basketball, it may be required that one action, like running, is inhibited and another action, such as throwing the ball, is executed when you notice that a player from the opposing team is approaching.

**Figure 1:**
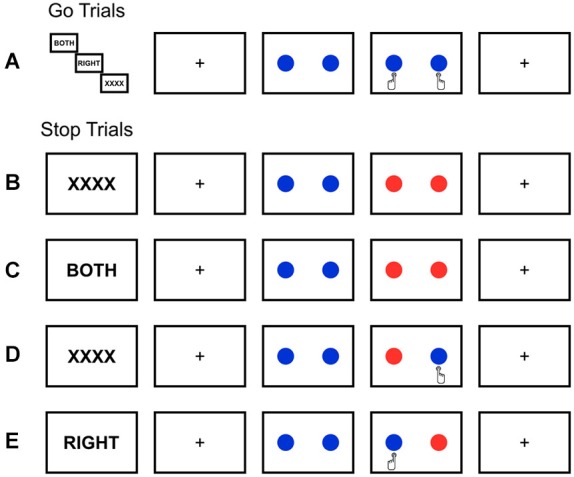
**Schematic overview of the modified stop-signal task. (A)** Go trials always required simultaneous bilateral responses, regardless of the condition or cue. Stop trials by condition **(B)** Global Reactive, **(C)** Global Proactive, **(D)** Selective Reactive, **(E)** Selective Proactive, whereby blue circles refer to go-stimuli and when they change to red, stopping is required. Cues indicate where the stop-signal will appear during proactive (i.e., “BOTH”; **C** and **E**) conditions or will not provide additional stopping information, as in the reactive conditions (i.e., “XXXX”; **B,D**). Location of the stop-signal indicates that either global (that is, bimanual; **B,C**) or selective (that is, unimanual; **D,E**) stopping is required. Furthermore, in selective conditions **(D,E)**, a successful stop trial is characterized not only by successful response inhibition to the stop-signal, but also by correct response execution of the other hand.

Thus, given these two modes through which cognitive control (Braver, 2012) may be operated and the global or selective nature of stopping behavior, a purely reactive explanation of inhibition represents a model that is poorly generalizable and provides little relevance for application, especially in the clinical realm (Aron, 2011). Until now, no paradigm has been developed which is suited to assess both manipulations of preparation (reactive vs. proactive) and selectivity (global vs. selective); thus, we developed and present such a paradigm within this paper (Figure 1).

To identify regions and networks contributing to response inhibition, neuroimaging methods, particularly functional magnetic resonance imaging (fMRI), have been employed. The right inferior frontal cortex (rIFC) seems to be critical in inhibiting responses and may act as a type of “brake” in conjunction with basal ganglia networks, which are critical for canceling movements (Aron et al., 2004, 2014; Aron, 2011). A cortical network which is used for reactive stopping, involving the presupplementary motor area (preSMA), rIFC and subthalamic nucleus (STN), may also be used in proactive inhibitory control if subjects slow down their responses in preparation of stopping (Chikazoe et al., 2009; Jahfari et al., 2011); while, the stopping mechanism involved (Aron, 2007, 2011) may be global via the hyperdirect pathway (Nambu et al., 2002) or selective via the indirect pathway (Smith et al., 1998), depending on the characteristics of the task design (Jahfari et al., 2011). Although recent strides have been made in identifying networks contributing to inhibition processes under various experimental manipulations, it is difficult with fMRI as a neuroimaging technique to make claims about when such neurophysiological changes occur in relation to the stopping process, given the rather poor temporal resolution of fMRI.

The relevance of the temporal electrophysiological characteristics of response inhibition has been widely demonstrated in the literature, especially when it comes to event-related potentials (ERPs) such as the occurrence of the N2/P3 complex in response to stop trials. The N200, which likely represents cognitive processes such as conflict monitoring is characterized by a strong theta response when subjected to time-frequency decompositions (Enriquez-Geppert et al., 2010; Yamanaka and Yamamoto, 2010; Schmiedt-Fehr and Basar-Eroglu, 2011); whereas, the P300 likely reflects response/inhibition-related processing or evaluation and demonstrates strong delta activity (Huster et al., 2013). This dissociation among processes has further been demonstrated in a go/no-go task, whereby a time-frequency decomposition of the N2/P3 complex was explained by theta and delta activity, respectively, and further supported the view others have made (Bernat et al., 2011; Huster et al., 2013) that these measure index separable processes (Harper et al., 2014). Increased delta and theta power for successful as compared to failed stopping (Wessel and Aron, 2013), as well as for stopping as compared to going (Lavallee et al., 2014) have also been reported in a similar temporal window. Furthermore, in a study comparing the go/no-go and continuous performance task (CPT), results suggested that delta activity reflected the demanding sustained attention requirement of the CPT (Kirmizi-Alsan et al., 2006). This finding is especially relevant considering that the CPT is actually a cued paradigm, which provides some basis for comparison with our paradigm (see Figure 1), which also involves constant cueing. In addition to the delta and theta power differences, high resolution electrocorticography (ECoG) recordings in patients demonstrated increased beta band power for successful vs. failed stop trials in a time period before the stop-signal reaction time (SSRT; Swann et al., 2011). Furthermore, studies also reported augmented beta power with successful inhibition at frontal (Alegre et al., 2008) and central EEG scalp sites (Krämer et al., 2011), suggesting that inhibitory control may be associated with oscillatory beta activity in a fronto-basal ganglia network (Aron, 2011). In addition to time-frequency decompositions of data, EEG studies employing other measures such as group independent component analysis (ICA) and Bayesian network estimations (Huster et al., 2014) and coherence measures (Anguera et al., 2013; Greenhouse and Wessel, 2013) demonstrate the utility of using EEG methods within the stop-signal paradigm. Of particularly high relevance for illustrating the importance of temporal effects within the stop-signal paradigm, utilizing stopping-related connectivity, Huster et al. (2014) present a previously undetected pattern of results, which delineate the relevance of exploring time-windows outside of the typical ERP/time-frequency peaks.

Considering that factors such as the selectivity (that is, global or selective) and preparatory control mechanisms (that is, reactive or proactive) employed during inhibition may have wider validity as an experimental model for stopping than simply observing reactive control of inhibition, a novel paradigm (Figure 1), utilizing uni (selective)- and bimanual (global) stopping in conjunction with neutral (reactive) and helpful (proactive) cues, was developed to incorporate these factors, and EEG methods were applied to capture the associated temporal nuances in oscillatory activity. Given the strong association between delta oscillations and the P300 in cognitive tasks (Basar-Eroglu et al., 1992; Demiralp et al., 2001; Ergen et al., 2008), and the role of P300 in attention and context updating (for a review see Polich, 2007), we claimed that delta activity should be modulated in case the preparatory mode shapes attention and behavior as suggested within the DMC framework (Braver, 2012), as well as by other authors (Aron, 2011). Based on the links of theta with conflict monitoring processes and the rather unexpected nature of reactive stopping, we expected elevated theta activity to be observed under reactive, as compared to proactive stopping conditions. Beta activity has been implicated in inhibitory processes (Swann et al., 2009), motor behavior and cognition (Engel and Fries, 2010); thus, we hypothesized that motoric beta effects should be observed in the contralateral motor cortex with respect to the stopping condition. That is, when stopping is required from the right hand, a decreased attenuation of motor beta activity should be observed in the left motor cortex (“relevant” for stopping) as compared to the right motor cortex (“irrelevant” for stopping); whereas, no significant difference should be observed for global (i.e., bimanual) stopping behavior. The question was originally posed as to whether proactive stopping could also be executed selectively as opposed to just globally, and a proactive selective set via the indirect pathway has been proposed (Aron, 2011); however, the electrophysiological associations are yet to be tested, especially in one cohesive paradigm which would allow for direct comparisons of the proactive and selective stopping to the reactive and global equivalents. Within this paper we demonstrate that delta activity reflects an EEG marker sensitive to both manipulations of selectivity and preparatory control.

## Materials and methods

### Participants

Twenty healthy participants were recruited from a database of subjects regularly participating in psychology/neuroscience experiments at the University of Oldenburg. All subjects (*n* = 20; 10 female; mean age = 24.63, SD = 2.47) were right-handed according to the Edinburgh Handedness Inventory and none of the subjects reported personal history of psychiatric or neurological disorders. All subjects had normal or corrected to normal vision. All participants provided written informed consent prior to participating, and the study was conducted in accordance with the Declaration of Helsinki and was approved by the local ethics committee of the University of Oldenburg.

### Experimental design

A modified stop-signal paradigm was implemented (Figure 1), which was designed to test the interaction between the global and preparatory mechanisms contributing to inhibition. These two factors (that is, SELECTIVITY and PREPARATION) were manipulated by cueing subjects to the location of a possible upcoming stop-signal (i.e., proactive preparation as in Figures 1C,E) or omitting the cue and providing neutral text in place of the cue, in the case of reactive control as in Figures 1B,D under unilateral (i.e., selective stopping, Figures 1D,E) or bilateral (i.e., global, Figures 1B,D) stopping conditions. Cues, displayed for 2 s, were presented anew every four trials and were always valid, such that the cue always applied to the upcoming consecutive four trials. On reactive trials whereby the cue was “XXXX” stop-signals did not necessarily appear in the same location if they occurred twice in one block of four consecutive trials. Given that the cues were always valid, the appearance of a stop-signal at any location following the presentation of an “XXXX” cue was possible (and henceforth valid); thereby, retaining the reactive nature of this condition. The stimuli in this visual stop-signal task were comprised of bilateral go-signals (white circles) equidistantly positioned around a central fixation cross and either uni or bilateral stop-signals (red overlay on original go-signal stimuli) on a black background, which in a subset of trials succeed the go-signal. Each trial began with a centrally positioned fixation cross for a randomly jittered duration (0–1000 ms), followed by the go-signals with a 550–900 ms duration in the case of go-trials; whereas, go-signals were presented for 250–600 ms before a stop-signal appeared, depending on performance, in the case of stop-trials. The stop-signal delay (SSD), the time between go- and stop-signals on stop-trials, was tracked independently for each condition and altered via a staircase method by adding or subtracting 50 ms to the initial 250 ms delay in the case of successful or failed inhibition, respectively, to achieve a 50% response rate (RR). Lower and upper boundaries for the SSD tracking procedure were set to 50 ms and 600 ms, respectively. The experiment, lasting 49 min in duration, was divided into four blocks (252 trials each) with 2-min pauses separating each and consisted of a total of 1008 trials, 33% of which were stop-trials. All go-trials, regardless of condition, required a simultaneous bilateral button response. Half of all stop-trials demanded global stopping (i.e., bilateral), whereas the selective stopping (i.e., unilateral) trials were equally distributed across left- and right-handed stopping. This design allowed us to directly test the main effects of SELECTIVITY (selective vs. global) and PREPARATION (reactive vs. proactive), as well as their interactions, on inhibition in one paradigm. Following every 36 trials, subject received feedback based on both reaction time and stopping accuracy. If average go reaction-times (goRTs) were longer than 600 ms or if RRs were above 50%, subjects received feedback to react faster (“Respond quicker”) or more accurately (“Respond more accurately”), respectively. If both reaction times were too long and RRs were too high, feedback was given that subjects should respond both faster and more accurately; otherwise, positive feedback was given (“Well Done”).

### EEG recording and preprocessing

Electroencephalogram was recorded from 64-channel electrode cap, placed in accordance with the 10–10 system for electrode placement and an additional electrooculogram (EOG) electrode, placed below the left eye, was used in order to record eye artifacts. Signals were amplified with a Brain Products amplifier (BrainAmp Plus) and digitized at 1000 Hz. Utilizing EEGLAB (Delorme and Makeig, 2004) as implemented in MATLAB, EEG data were low-pass filtered at 35 Hz and re-referenced to the common average reference and down-sampled to 250 Hz. In order to enhance the signal to noise ratio, and to allow for the possibility of a single-trial analysis, the data were decomposed by means of a temporal ICA (extended infomax) and components representing muscle or eye artifacts were removed, as this is a well-suited method for EEG artifact detection and correction (Delorme et al., 2007).

### EEG time-frequency analysis

Time-frequency decompositions were computed using functions provided by the EEGLAB open source software. Frequencies from 0 to 35 Hz were analyzed using 100 frequency steps. The upper and lower boundaries for baseline correction were, respectively, −800 and −100 ms before stimulus onset. Power values for each time-frequency bin from stimulus-onset (0 ms) until 1214 ms post stimulus-onset were normalized by dividing the frequency-specific power during baseline. Subsequently, event-related spectral perturbation (ERSP) values were calculated by performing log transform from all electrode positions, resulting in dB values for the time-frequency data (Delorme and Makeig, 2004; Grandchamp and Delorme, 2011). These mean ERSP-values were extracted from electrode sites of interest (EOIs) within temporally specific windows for different frequency bands (for a tutorial on the topic, see Herrmann et al., 2014). Post-stimulus onset time-windows were defined post-go-stimulus for go-trials and post-stop-stimulus for stop trials. Delta (0–4 Hz) values were extracted between 300 and 400 ms post-stimulus onset; whereas, theta (4–8 Hz) values were extracted between 150 and 250 ms post stimulus-onset (See Figures 3A,B, left panels). These time ranges were chosen to correspond to the typical temporal progression of delta/theta activity patterns and the N2/P3 ERP complex observed in stop-signal paradigms (Huster et al., 2013). Furthermore, high-beta (21–30 Hz) values were extracted between 220 and 500 ms post stimulus-onset, in correspondence with previous work (Ritter et al., 2009; Swann et al., 2009, 2011).

**Figure 2:**
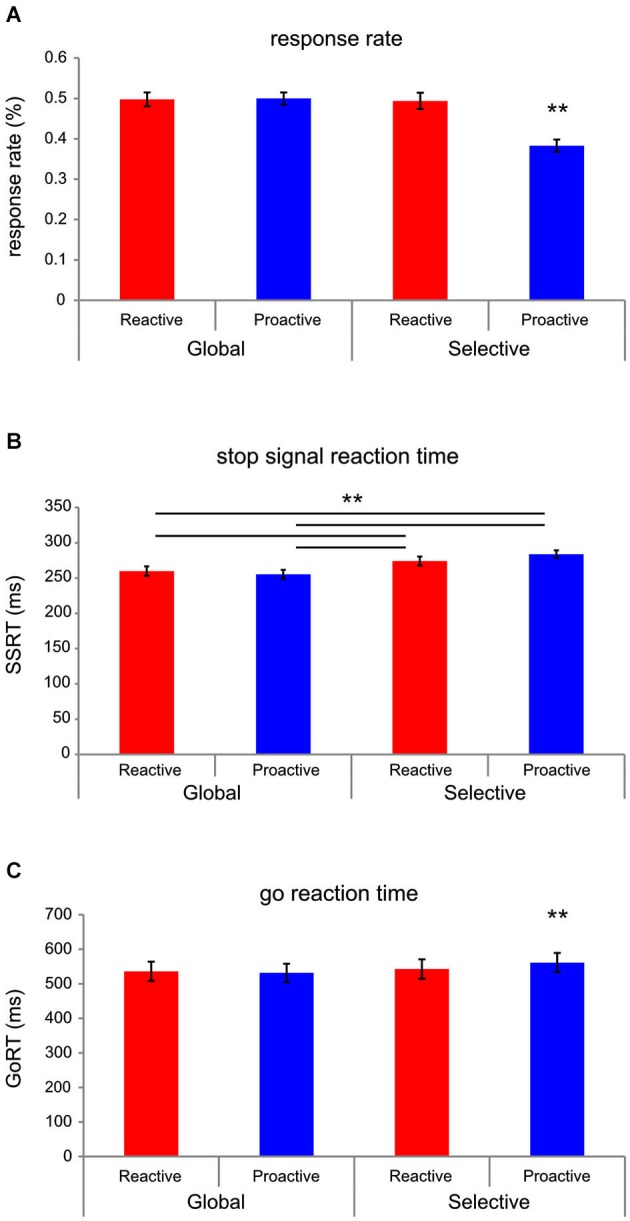
**Behavioral results**. Significant differences for **(A)** response rate (RR), indicating the rate of unsuccessful inhibition, **(B)** stop-signal reaction time (SSRT), an estimate of stopping latency, and **(C)** go reaction time (goRT) are presented. ** *p* < 0.01. Under proactive selective stopping conditions, the RR is significantly lower and goRT is significantly higher in comparison to all other conditions. Please refer to Table 1 for a complete list of means and SEMs.

**Table 1:** **Behavioral results**.

	Reactive global	Proactive global	Reactive selective	Proactive selective
goRT	534.62 (29.15)	530.18 (27.85)	541.01 (29.55)	559.55 (29.12)
RR	0.497 (0.018)	0.4988 (0.015)	0.4905 (0.021)	0.3854 (0.015)
SSRT	255.29 (5.05)	252.31 (5.92)	270.38 (5.31)	280.83 (4.62)
SOA	279.34 (29.58)	270.62 (27.14)	277.87 (28.07)	278.72 (26.06)
Stopping interference			125.59 (9.75)	79.43 (9.41)

*Mean (standard error) for goRT, RR, SSRT and SOA across the four conditions, as well as the stopping-interference RT measure for reactive and proactive stopping under selective conditions*.

**Figure 3:**
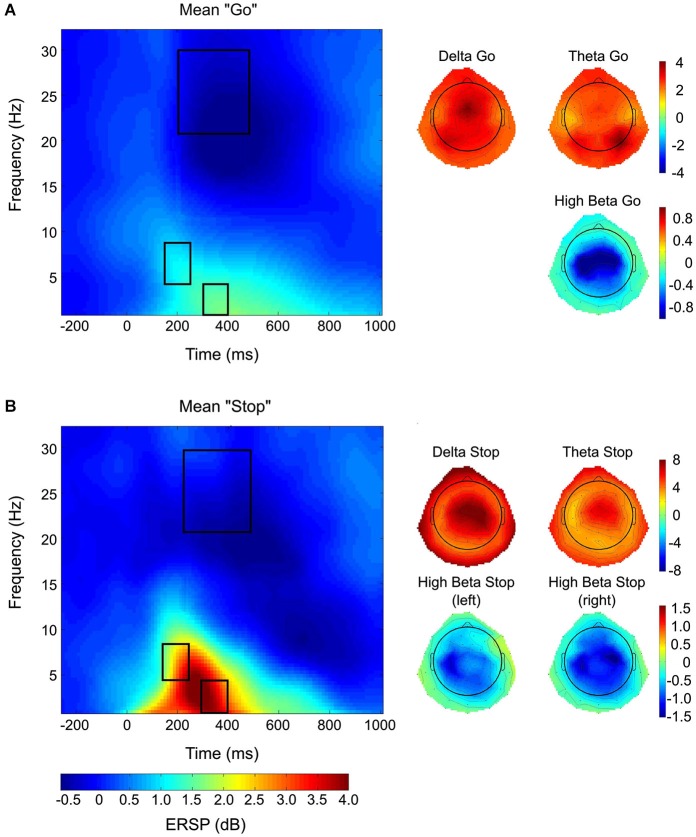
**Time-frequency plots and topographies**. Time-frequency plots averaged across go **(A)** and stop **(B)** conditions (left panels), taken from electrode Cz, are presented, as well as corresponding topographies for the frequency-bands of interest (right panels). Delta and theta activity, which demonstrate centralized topographies, are significantly elevated in the stopping conditions as compared to going conditions. Furthermore, lateralized high-beta activity during stopping demonstrates stronger lateralization for left-sided stopping as compared to right-sided stopping, as further demonstrated statistically (See Figure 4D).

**Figure 4:**
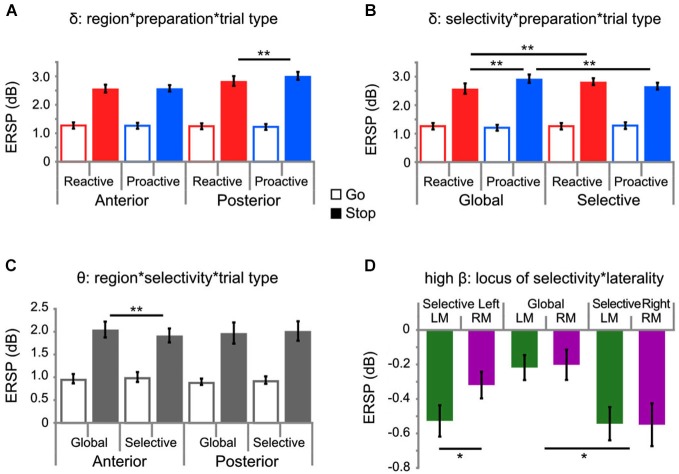
**EEG results**. Results from analysis of EEG time-frequency data showing significant interactions for delta **(A,B)**, theta **(C)**, and high-beta **(D)** activity, whereby * *p* < 0.05 and ** *p* < 0.01. For panels **4A–C**, go-trials are depicted by open bars and stop-trials are depicted by closed-in bars, in terms of the color shading. Panel **4D** depicts only stop-trials. Note that horizontal bars indicate significant sources of interactions. A regional delineation in delta **(A)** and theta **(C)** band activity by preparation and selectivity, respectively, was observed; whereas, only activity in the delta band was modulated by both manipulations to preparation and selectivity **(B)**. This modulation **(B)** showed no regional specificity and the results presented represent values pooled across all EOIs. Stop-related activity in the high-beta band only showed significant motor lateralization under selective stopping with the left hand **(D)**.

The aforementioned time-frequency features were extracted for defined EOIs, whereby the mean activity over single electrodes (specified below) was calculated. Given the regional specificity of inhibition and cognitive control related processes in the brain, lateralized (left/right) anterior (frontal) and posterior (parietal), as well as central (reflecting motor activity, when lateralized) EOIs were used for statistical analyses. These EOIs included frontocentral (F1, F2, FC1, FC2, FCz, Cz) and centroparietal (C1, C2, CP1, CP2, CPz, Pz) regions, which correspond to regions displaying increased delta and theta band activity during stop-signal tasks (Huster et al., 2013). Given the role of beta band activity in frontal and motor regions during inhibition (Swann et al., 2009), frontal and motor cortex regions were also statistically assessed. Specifically, the left (F7, F5, AF3, AF7) and right (F6, F4, AF4, AF8) inferior frontal cortices, as well as the left (C5, C3, CP3) and right (C4, C6, CP4) motor cortices were identified as EOIs.

### Statistical assessment

Behavioral features of the stop-signal task, such as go reaction time (goRT), RR, stopping-interference and SSRT were analyzed with repeated measures analyses of variance (ANOVA) with SELECTIVITY (global, selective) and PREPARATION (reactive, proactive) as within subject factors. The RR measure, which represents the percentage of unsuccessful stop trials, is computed as the number of failed stop trials divided by the number of all stop trials and can be considered a measure of stopping accuracy. That is, the lower the value, the more successful participants were at correctly inhibiting responses on stop trials. The SSRT measure, inherently unique to the stop-signal task, is estimated based on the horse-race model of stopping, which describes stopping and going processes as racing for the first finishing time. These competing processes have been used to describe stop-signal task performance (Logan et al., 1984). Given that the stopping mechanism itself cannot be directly measured, SSRT must be estimated to derive the proposed time required for stopping a response. The SSRT was assessed as the difference between mean goRT and the average stimulus onset asynchrony (SOA), which is the time between the go and stop signal on stop trials (Band et al., 2003). Furthermore, the stopping—interference effect was assessed under conditions of selective stopping (that is, when unimanual stopping was required under proactive and reactive contexts). Under selective stopping conditions, participants are required to stop one response (i.e., with the right index finger) and continue execution of the alternative response (i.e., left index finger) on stop trials (Coxon et al., 2007). Delays in the execution of the alternative response on stop-trials may provide insight into the underlying stopping mechanism (Coxon et al., 2006). Thus, the stopping-interference effect was calculated as the difference between execution of the alternative response on selective stop trials and their matched go-trials. When statistical assessments were conducted separately for response hands, no significant differences were revealed; therefore, behavioral data averaged across response hands will be reported.

Stimulus-related time-frequency EEG data (ERSP) from theta and delta bands were analyzed with the use of repeated measures ANOVAs, with factors SELECTIVITY (global, selective), PREPARATION (reactive, proactive), TRIAL-TYPE (stop, go), REGION (anterior, posterior) and LATERALITY (left, middle, right) as within-subject factors. Furthermore, to test the specific hypotheses regarding the effects of selectivity (i.e., left- and right-handed stopping vs. global stopping) and corresponding motor cortex beta effects, time-frequency EEG data from the high-beta band were analyzed with a repeated measures ANOVA, with factors PREPARATION (reactive, proactive), LOCUS of SELECTIVITY (Global, Selective Left, Selective Right) and MOTOR LATERALITY (Left, Right) for successful stopping only. The LOCUS of SELECTIVITY factor refers to whether stopping was required from both hands (Global), or only the left (Selective Left) or right (Selective Right) side; whereas the MOTOR LATERALITY factor refers to the lateralization of the motor cortex EOI, such that we are reporting activity from either the left or right motor cortex. When appropriate, *post hoc* testing was conducted using the Tukey Honestly Significant Different (HSD) method. Tests of the previously specified hypotheses were assessed within the omnibus models using planned comparisons.

**Figure 5:**
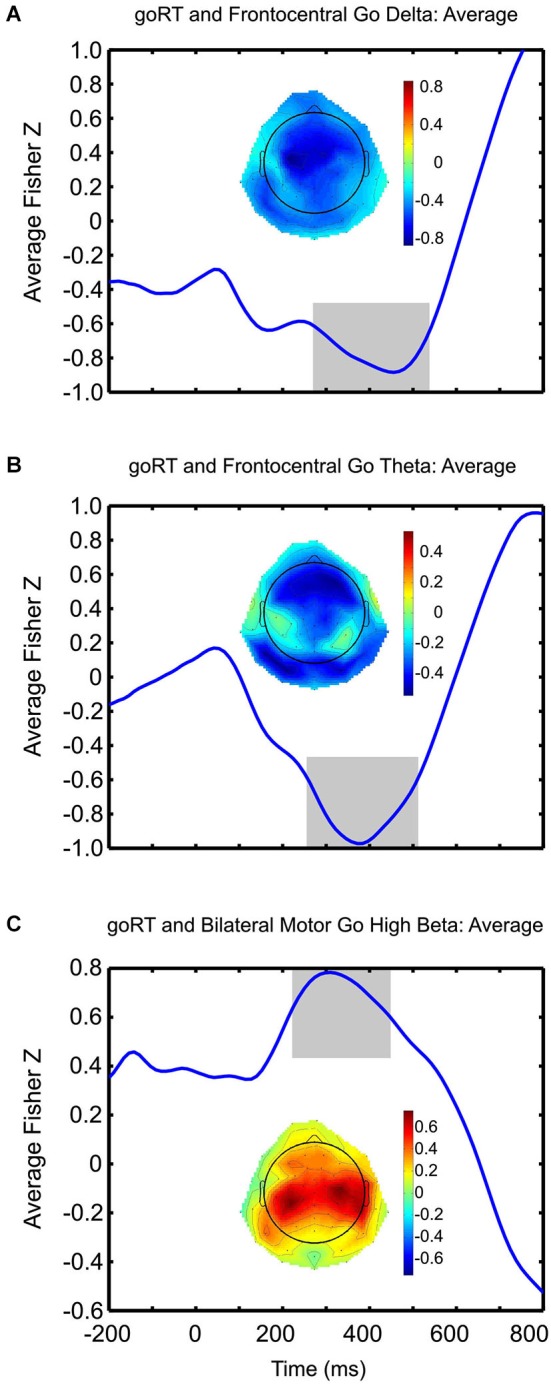
**Brain-behavior correlations**. Average Fisher-z transformed correlations between goRT and go-related frontocentral delta **(A)**, theta **(B)** and bilateral motor high-beta **(C)** activity are plotted over time. Within each plot, the topographies depict correlation strength between goRT and associated EEG activity for each scalp-electrode. Negative correlations, observed in a fronto-central topography, between goRT and delta and theta activity are strong and peak rather late, with respect to typically observed N2/P3-like effects; whereas, strong positive correlations between goRT and high-beta activity are observed over the motor cortices and peak just after 300 ms post-stimulus onset. The areas shaded in gray represent the time periods where significant correlations were observed.

Further statistical analyses, which were exploratory in nature, were employed to uncover regionally specific correlations between stimulus-related time-frequency data and behavioral measures. With these exploratory correlations, we naturally adopted a more liberal approach to interpreting the statistical effects. Pearson correlations between goRT and delta, as well as theta and high-beta activity were calculated across all electrode channels for each condition, separately, and plotted topographically, based on electrode location. The effects observed were very similar and did not vary between conditions. Given the highly overlapping pattern of results across the conditions, a Fisher z-transformation of the data was performed (Everitt, 2002) so that we could average Pearson correlation coefficients across conditions in order to avoid presenting redundant results across conditions. The Pearson correlation coefficients were Fisher z-transformed before plotting, averaged over conditions and plotted at each electrode site (Figure 5). To further examine if these correlations were bound to a temporal range, such as those time-windows previously identified as relevant (see: Section Materials and Methods, EEG time-frequency analysis), correlations between delta, theta, as well as high-beta activity and goRT were computed and plotted across time, whereby we show the average over all conditions in Figure 5.

## Results

### Behavioral results

An interaction among SELECTIVITY and PREPARATION demonstrated that RRs were significantly lower under proactive selective stopping conditions (*F*_(1,19)_ = 36.44, *p* < 0.001, partial *η*^2^ = 0.591; see Figure 2A). A main effect of SELECTIVITY (*F*_(1,19)_ = 14.824, *p* < 0.005, partial *η*^2^ = 0.438) was observed for SSRT, whereby SSRTs were longer under the selective (*M* = 275.61, SEM = 4.41), as compared to the global (*M* = 253.78, SEM = 4.835) stopping condition. Furthermore, under the condition of proactive selective stopping, SSRTs were significantly longer in comparison to all other conditions (*F*_(1,19)_ = 7.044, *p* < 0.05, partial *η*^2^ = 0.27; see Figure 2B) except for reactive selective stopping, as evidenced by an interaction between SELECTIVITY and PREPARATION. An interaction among SELECTIVITY and PREPARATION indicated that goRTs were significantly higher under this same condition (*F*_(1,19)_ = 9.339, *p* < 0.01, partial *η*^2^ = 0.33; see Figure 2C) compared to all other conditions indicating a different behavioral strategy. Stimulus onset asynchronies, which were tracked dynamically throughout the experiment, did not differ significantly across conditions. Under conditions of selective stopping, we further tested for the stopping interference effect (Aron and Verbruggen, 2008), which assessed the difference in RTs on go-trials and their concomitant selective stop-trials (i.e., execution of alternative response) during the selective stopping conditions, under both reactive and proactive control. Again, left- vs. right-handed differences were not observed (*p* > 0.7). We did, however, observe a larger stopping-interference effect under reactive control conditions, as compared to proactive control conditions (*F*_(1,19)_ = 49.32, *p* < 0.0001, partial *η*^2^ = 0.722; See Table 1).

### EEG results

Average time-frequency plots from electrode Cz are shown for go (Figure 3A, left panel) and stop (Figure 3B, left panel) conditions, with the corresponding time-windows of interest (see Section Materials and Methods) outlined in black. Topographies are shown for frequency-bands of interest, averaged across all go-conditions (Figure 3A, right panel). For the averaged stopping conditions, we presented topographies for delta, theta, as well as high-beta under lateralized stopping conditions (Figure 3B, right panel).

#### Delta

A significant three-way interaction was observed between factors SELECTIVITY, PREPARATION, and TRIAL-TYPE (*F*_(1,19)_ = 22.212, *p* < 0.001, partial *η*^2^ = 0.539). *Post hoc* testing revealed increased delta activity under conditions of global proactive stopping (*M* = 2.929, SEM = 0.114), as compared to global reactive (*M* = 2.582, SEM = 0.18, *p* < 0.0005) and selective proactive stopping (*M* = 2.667, SEM = 0.121, *p* < 0.0005; Figure 4B). Furthermore, elevated delta activity was observed during selective reactive stopping (*M* = 2.8235, SEM = 0.121) in comparison to global reactive stopping (*p* < 0.05). An additional interaction involving REGION, PREPARATION and TRIAL-TYPE was observed for delta activity (*F*_(1,19)_ = 15.364, *p* < 0.001, partial *η*^2^ = 0.447; Figure 4A), whereby increased delta was observed during proactive stopping (*M* = 3.016, SEM = 0.14) as compared to reactive stopping (*M* = 2.58, SEM = 0.113, *p* < 0.001) in posterior regions; however, these differences are not significant in anterior regions (*p* = n.s.).

#### Theta

In the theta band, a significant three-way interaction between factors REGION, SELECTIVITY and TRIAL-TYPE (*F*_(1,19)_ = 25.014, *p* < 0.0001, partial *η*^2^ = 0.568) was observed, whereby *post hoc* testing revealed that the source of the interaction was only occurring in anterior electrode regions (Figure 4C). *Post hoc* testing via Tukey’s HSD revealed higher theta activity under conditions of global stopping, as compared to selective stopping in anterior regions (*p* < 0.0005), but not in posterior regions (*p* = n.s.).

#### High beta

Within the high-beta band, a significant interaction between LOCUS of SELECTIVITY and MOTOR LATERALITY was observed (*F*_(2,18)_ = 3.92, *p* < 0.05, partial *η*^2^ = 0.303; Figure 4D) during inhibition (i.e., only stop-trials considered in this model), whereby only under conditions where selective stopping was required from the left hand, was the contralateral (i.e., right) motor cortex activity (*M* = −0.3194, SEM = 0.077) more positive (i.e., less negative) than the ipsilateral (i.e., left) motor cortex activity (*M* = −0.5273, SEM = 0.0908; *p* < 0.001). Further *post hoc* testing revealed significantly (*p* < 0.0001) attenuated left motor beta activity under conditions of selective right-handed stopping (*M* = −0.5436, SEM = 0.0964), as compared to left motor beta activity under conditions of global stopping (*M* = −0.2179, SEM = 0.0724). The PREPARATION factor did not significantly modulate high-beta activity.

### Brain-behavior correlations

Correlation coefficients after Fisher z-transformations are plotted topographically for each electrode site, showing stronger correlations between goRT and go-signal high-beta activity concentrated over the motor cortices, bilaterally (Figure 5C). Averaged over conditions, the temporal fluctuations of these correlations show the strongest relationship (Fisher z-transformed *r* = 0.7833, *p* < 0.001), peaking at 310 ms, but were significant between 224 and 444 ms (*p* < 0.05, uncorrected) post stimulus onset. Although correlations between delta activity and goRT showed a more diffuse pattern (Figure 5A), correlations across conditions seemed to cluster around central sites, with peak negative correlations (Fisher z-transformed *r* = −0.8842, *p* < 0.001) occurring at 456 ms post stimulus onset, whereby correlations were significant (*p* < 0.05, uncorrected) from 286 to 530 ms post-stimulus. Correlations between theta activity and goRT demonstrated a rather fronto-central topography (Figure 5B), whereby across conditions, correlations (Fisher z-transformed *r* = −0.973, *p* < 0.001) peaked at 372 ms post-stimulus and were significant between 262 and 506 ms post-stimulus. Furthermore, a negative correlation between SSRT and stop-related fronto-central theta activity in the global stopping condition (*r* = −0.41, *p* < 0.05) was observed occurring at roughly 200 ms post-stimulus onset that was not found for the selective stopping conditions.

## Discussion

The majority of studies conducted with the stop-signal paradigm focus on the reactive control of inhibition, as probed with the classic version of this task and only few studies assess how context, preparation and selectivity influence the stopping process (Aron and Verbruggen, 2008; Cai et al., 2011; Lavallee et al., 2014). To date, no studies have been conducted whereby the task employed provided the opportunity to test for interacting effects of preparation and selectivity on the stopping process within one coherent paradigm. Although one possible limitation of the paradigm is that under reactive conditions, after the appearance of the first stop-signal, one may argue that the reactive nature of this condition is somewhat reduced. We have countermanded this insofar as that if a second stop-signal appears on a reactive trial that it does not necessarily occur in the same location; thus retaining the reactive nature of this condition. Furthermore, only few EEG studies conducted more recently have examined stop-related effects with respect to the frequency domain (Krämer et al., 2011; Nigbur et al., 2011; Schmiedt-Fehr and Basar-Eroglu, 2011; Wessel and Aron, 2013; Lavallee et al., 2014), as opposed to focusing mainly on ERPs (Huster et al., 2013). Therefore, to provide further insight into a richer model of stopping, we conducted the first study, which assessed the temporal oscillatory dynamics of stopping under both manipulations to the preparatory control and selective nature of the stopping behavior. Along these lines, a number of interesting observations were made with respect to oscillatory changes (Figure 4), as well as behavioral differences (Table 1) and we further demonstrate temporal patterns of brain-behavior correlations otherwise not reported in the literature (Figure 5).

Behavioral results (see Table 1) indicate fewer false alarms, longer goRTs and prolonged SSRTs under conditions of proactive selective control. Also, in comparison to reactive selective stopping conditions, proactive selective stopping was associated with a shorter stopping interference effect. The SSRT results support predictions made by Aron and Verbruggen (2008). That is, subjects required significantly more time to stop an already initiated response when they had been cued as to which response may have to be stopped under selective, but not under global conditions. Thus, foreknowledge of which response(s) to prepare to stop engages a different stopping mechanism in both the global (bimanual) and selective (unimanual) stopping conditions. These results show parallels to the selective modulation of activity within the theta band. Increased theta was observed for global as compared to selective stopping (See Figure 4D) and subjects demonstrated faster SSRTs under this condition, which, physiologically speaking, may reflect increased interregional communication under global stopping conditions (Jensen and Colgin, 2007); thereby supporting faster speeds at which stopping can be successfully executed. Indeed, negative correlations between stop-related theta activity and SSRTs were observed in the global stopping conditions occurring roughly 200 ms post-stimulus onset; such associations were otherwise not found under selective stopping conditions. The prolonged SSRTs under proactive selective conditions and the less costly stopping interference effects under proactive conditions support the hypothesis that subjects will use a selective mechanism when provided helpful foreknowledge (proactive) regarding which response must be stopped, consistent with that of the work presented by Aron and Verbruggen (2008). According to Braver (2012), one of the advantages of a proactive mode of control is optimization of preparation while minimizing sources of distraction, which may be reflected in the reduced number of errors and lower stop-interference delay in this proactive selective condition; however, this proactive mode of control is not without its disadvantages. The proactive control strategy may be relatively more resource consuming in comparison to reactive control modes, and this disadvantage may be further reflected in prolonged SSRTs as compared to reactive control conditions. Whereas, under reactive conditions, goal representations are only accessed at a time in which they are needed (hence, costlier stop-interference delays), the constant maintenance of goal representations in proactive settings may require a heavier cognitive load.

In correspondence with the work of others (Huster et al., 2013; Wessel and Aron, 2013; Lavallee et al., 2014), increased delta and theta activity in a post-stimulus window was observed for successful stop-trials, as compared to go-trials; moreover, a regional delineation of delta and theta activity by preparation and selectivity, respectively, was observed (See Figures 4A,C). A three-way interaction involving REGION, TRIAL-TYPE and PREPARATION (See Figure 4A) which demonstrated increased delta activity observed during proactive stopping, as compared to reactive stopping in posterior electrode sites may reflect the use of posterior attentional network (Petersen and Posner, 2012). The relationships between delta activity, the P300 and attentional mechanisms, such as context-updating (Donchin and Coles, 1988) and processing task-demands (Kok, 2001) have already been outlined in the literature (Basar-Eroglu et al., 1992; Demiralp et al., 2001; Polich, 2007). Additionally, by means of comparing delta activity during a go/no-go and CPT task, the role of delta activity was implicated in the sustained attention requirements of the CPT. If delta activity occurring within the time-range of the N2/P3 complex is reflective of sustained attention, as previously suggested (Kirmizi-Alsan et al., 2006), then the results presented in Figure 4A may support this idea due to the increased delta activity for proactive conditions as compared to reactive conditions. That is, under proactive conditions the sustained attentional efforts would be comparatively higher than under reactive conditions, as the subject has to maintain the goal-relevant information (provided via the cue) and adjust attention accordingly. Thus, the use of such a delta network demonstrates that the proactive feature of this specific stopping condition steers attentional focus and thus reflects the goal-oriented constitution of proactive control, in contrast to the abrupt, deer in the headlights nature of reactive control (Aron, 2011; Braver, 2012).

Furthermore, only delta activity was modulated by all factors (TRIAL-TYPE, SELECTIVITY, PREPARATION), whereby increased delta activity was observed during successful stop-trials under proactive global conditions, as compared to reactive global and selective proactive conditions (See Figure 4B). These results demonstrate that not only is a proactive selective stopping set possible, but also that important performance measures, such as the number of errors (RR) and goRTs, as well as SSRTs, are modulated significantly in this condition. Furthermore, the electrophysiological results indicate that the interaction between selectivity and the preparatory mode of stopping is mediated by delta activity. Intuitively, one may initially expect theta activity, rather than delta activity to be modulated by the preparation condition, as there should technically be less conflict due to the upcoming foreknowledge under proactive conditions as compared to reactive conditions; however, theta activity was not significantly modulated by the PREPARATION factor. Although surprising, this could be due to different possible theta networks within the brain and could rather point to the role of theta for interregional communication (Jensen and Colgin, 2007; Mizuseki et al., 2009), as we observed significantly elevated theta activity for global, as compared to selective stopping only at anterior electrode sites (See Figure 4C).

Stop-related effects observed in the high-beta band partially supported our hypotheses that there would be a significant modulation in motoric beta activity in the hemisphere contralateral to the stopping side (Figure 4D). Such a contralateral motoric beta modulation was only observed when selective stopping was required from the left hand. One may interpret this effect in the context of a dominant-hand explanation whereby significantly different levels of beta are not required for selective stopping of the dominant hand (all subjects right handed), but this modulation is, however, required for the non-dominant hand. Not only has a hemispheric asymmetry of motor cortex activation (Stancák and Pfurtscheller, 1997) and structure (Westerhausen et al., 2007) been reported, but also a hemispheric asymmetry of inhibition in right-handed subjects (Netz et al., 1995), although this may not be reflected in corticospinal fiber tract characteristics (Westerhausen et al., 2007). The left motor cortex, in a group of right-handed participants, exerts more effective inhibitory control (Ziemann and Hallett, 2001); thus, if the left motor cortex is more effective at inhibiting contralateral movements, perhaps the disparity between the two motor cortices is not as great for dominant-handed stopping, as it is for stopping required from the non-dominant hand (i.e., significantly different left and right motor cortex activation for selective stopping on the left side, Figure 4D). Interestingly, despite EEG differences in the modulation of motoric beta activity during selective stopping for the non-dominant as compared to the dominant hand, no significant differences in behavioral performance between left- and right- handed responses were observed.

Brain-behavior relationships with stimulus-related oscillatory activity and behavioral measures, although demonstrating no condition-wise specificity, reveal a clustering of positive correlations between goRT and go-trial related high-beta activity in the bilateral motor cortices (Figure 5C). Although these tests were not corrected for multiple comparisons, given the exploratory nature of our analyses, these results point to a possible region-wise modulation of the readiness of the motor system to execute a response, as a decreased attenuation of motor high beta activity (i.e., less negative ERSPs) for go-trials was associated with slower reaction times. Temporal fluctuations of these aforementioned correlation coefficients were assessed and revealed the peak maxima at 310 ms post-stimulus onset, which is within a time-window deemed temporally relevant for beta activity (Swann et al., 2009; Engel and Fries, 2010). Additional exploration within the delta and theta bands revealed the highest topographical density of correlations in central and fronto-central regions, respectively, indicating higher power in the delta and theta ranges was associated with faster goRTs (Figures 5A,B). Although these findings are in correspondence with others demonstrating a negative correlation between P300 and reaction times (Holm et al., 2006; Ramchurn et al., 2014), and given the close relationship demonstrated between P300 and delta activity (Basar-Eroglu et al., 1992; Demiralp et al., 2001; Ergen et al., 2008), the average peak correlations occurred rather late, 372 ms post-stimulus onset for theta and 456 ms for delta, thus rather suggesting performance monitoring processing. Furthermore, these results await replication and validation as we did not apply stringent tests for multiple comparisons, but rather assessed the temporal dynamics of brain-behavior relationships; thus, our interpretations, although made in the context of already well-defined behavioral and electrophysiological findings, should be considered starting points for future research. These differential temporal results demonstrate not only the relevance of brain-behavior associations in the stop-signal paradigm, but further support conclusions drawn by Huster et al. (2014) that a focus on only peak activity provides an incomplete understanding of effects.

## Conclusion

We have provided the first account of behavioral modulation of stopping measures under conditions manipulating the preparatory control and the selectivity of stopping. Moreover, we illustrate temporally specific changes in oscillatory activity related to these experimental manipulations. With the use of this novel paradigm, progress in understanding inhibition under important task manipulations has been made insofar as delta activity appears to be the prime time-frequency feature sensitive to both selectivity and preparatory manipulations. These results further contribute to a more encompassing and generalizable model of inhibition; however, research in this very active field should be continued to gather more evidence of electrophysiological changes within this more parsimonious model of inhibition. Despite the immense impact that discerning such features of inhibition will have for patients of many neuropsychological disorders and the relationship of impaired executive functions to clinical and social phenomena (Miyake and Friedman, 2012), it may also be just as fruitful in understanding normal development of inhibition in healthy aging (van de Laar et al., 2011, 2014; Vink et al., 2014).

## Conflict of interest statement

The authors declare that the research was conducted in the absence of any commercial or financial relationships that could be construed as a potential conflict of interest.
